# Short-Term Effects of the Serious Game “Fit, Food, Fun” on Nutritional Knowledge: A Pilot Study among Children and Adolescents

**DOI:** 10.3390/nu11092031

**Published:** 2019-08-30

**Authors:** Sophie Laura Holzmann, Hanna Schäfer, Georg Groh, David Alexander Plecher, Gudrun Klinker, Gunther Schauberger, Hans Hauner, Christina Holzapfel

**Affiliations:** 1 Institute for Nutritional Medicine, Else Kröner-Fresenius-Center for Nutritional Medicine, School of Medicine, Klinikum rechts der Isar, Technical University of Munich, 80992 Munich, Germany; 2 Research Group Social Computing, Department of Informatics, Technical University of Munich, 85748 Garching, Germany; 3 Chair for Computer Aided Medical Procedures & Augmented Reality, Department of Informatics, Technical University of Munich, 85748 Garching, Germany; 4 Chair of Epidemiology, Department of Sport and Health Sciences, Technical University of Munich, 80992 Munich, Germany

**Keywords:** serious game, gamification, nutritional knowledge, children, adolescents, Germany

## Abstract

“Serious games” are a novel and entertaining approach for nutritional education. The aim of this pilot study was to evaluate the short-term effectiveness of “Fit, Food, Fun” (FFF), a serious game to impart nutritional knowledge among children and adolescents. Data collection was conducted at two secondary schools in Bavaria, Germany. The gameplay intervention (gameplay group; GG) consisted of a 15-minute FFF gameplay session during each of three consecutive days. The teaching intervention (teaching group; TG) was performed in a classic lecture format. Nutritional knowledge was evaluated via questionnaires at baseline and post-intervention. Statistical analyses were performed using R (R Core Team, 2018). In total, baseline data were available for 39 participants in the GG and 44 participants in the TG. The mean age was 13.5 ± 0.7 years in the GG and 12.8 ± 0.9 years in the TG. There was a significant (*p*-value < 0.001) improvement in nutritional knowledge in both intervention groups. Moreover, a between-group difference with a significantly (*p*-value = 0.01) higher increase in nutritional knowledge was detected for the TG. This pilot study provides evidence for the short-term effectiveness of both educational interventions on the improvement in nutritional knowledge. Finally, the FFF game might be an adequate educational tool for the transfer of nutritional knowledge among children and adolescents.

## 1. Introduction

Globally, the prevalence of overweight and obesity among children and adolescents increased 10-fold from 11 million in 1975, to 124 million in 2016 [1]. In Germany, the prevalence of overweight and obesity among children and adolescents (3 to 17 years) is estimated at 15% and 6%, respectively, with increasing trends in later life [2]. In particular, school entry and school age are associated with a considerable increase in the prevalence of overweight and obesity. Therefore, established prevention programs (e.g., nutritional education) aim to address this health challenge. Besides these traditional approaches, “serious games” are novel digital tools for educational purposes. Serious games are games which are applied in non-gaming contexts [3], and have motivational and enjoyable characteristics [4,5,6]. Serious games for health-related behavioral change are becoming increasingly available [7,8,9,10,11]. There is evidence that serious games can enhance the long-term retention of information and can promote behavioral change maintenance [6,12]. Thus, serious games may have the potential to become a novel digital educational method to improve nutritional knowledge and behavior in an entertaining and intrinsically motivating format [9,12,13].

Literature indicates that nutritional education by serious games can be effective among children and adolescents [9,10,13]. To date, several studies have investigated the effects of gameplay on nutritional knowledge and/or dietary change among children and adolescents [4,14,15,16]. Schools are a popular setting for educational (e.g., game-based) interventions because of their large availability of children and adolescents. Moreover, a digital game might be an entertaining alternative to traditional classroom teaching [10,17]. According to systematic reviews, school-based dietary interventions (e.g., curriculum, fruit and vegetable distribution, and serious games) can improve diet-related outcomes among children and adolescents [18,19]. Furthermore, a recent review on nutritional education and games for dietary behavioral change revealed that the majority of publications (21 out of 22) reported positive effects of playing digital games on dietary behavior and nutritional knowledge [13].

Sharma et al. evaluated the effects of the computer game “The Quest to Lava Mountain” on dietary behavior among primary school students over six weeks. Compared to the control group, the game intervention group showed a significant decrease in sugar consumption (*p* = 0.021) from pre- to post-intervention [20]. However, participants played the game on average 274 ± 110 minutes (min) during the six weeks, which was less than the recommended weekly dosage of game exposure (90 min) [20]. A further study showed that playing the multimedia game “Squire’s Quest” for five weeks resulted in a higher fruit, juice, and vegetable consumption (1.0 serving) among fourth-grade students compared to the control condition [17]. Moreover, a randomized trial conducted by Baranowski et al. revealed that playing knowledge-based video games for two months resulted in a significant increase in children’s daily fruit and vegetable consumption compared to controls playing website-based nutritional knowledge games [21]. Finally, serious games might be an appropriate educational tool for imparting and promoting nutritional knowledge and behavior among children and adolescents (e.g., in schools) [10,16].

The aim of this pilot study was to evaluate the short-term effectiveness of the serious game “Fit, Food, Fun” (FFF) on nutritional knowledge among secondary school students. Additionally, the change in nutritional knowledge was compared between the game-based approach and a traditional classroom teaching (classic lecture) format.

## 2. Materials and Methods

### 2.1. Study Participants and Design

The pilot study was conducted in two secondary schools in the city and district of Rosenheim (Bavaria, Southern Germany). According to the information given by the schools, the proportion of boys (67%) was higher than the proportion of girls. Teachers forwarded the study invitation to all 7th and 8th grade students for voluntary participation. Three classes (two 7th grades, one 8th grade) participated in each school. The schools were assigned to either the gameplay (GG) or the teaching (TG) intervention group. All 7th and 8th grade students with sufficient German language skills and written parental consent were eligible to participate. The study was approved by the Ethical Committee of the School of Medicine, Technical University of Munich (number: 175/18 S) and by the Rosenheim school board (Bavaria, Germany).

### 2.2. Intervention

The educational content of both interventions was based on the “10 guidelines of the German Nutrition Society for a wholesome diet” (https://www.dge.de/ernaehrungspraxis/vollwertige-ernaehrung/10-regeln-der-dge/en/). In total, six guidelines (rules) were selected, which referred to the recommended intake of whole-grain foods (1), sugar and salt (2), health-promoting fats (3), vegetables and fruits (4), and water (5), as well as to sports and physical activity (6). Two rules per day were displayed during gameplay or were written on the blackboard during the teaching session.

#### 2.2.1. Gameplay Group

The FFF game was designed as a serious game to impart nutritional knowledge in an entertaining format. The game was developed by an interdisciplinary research group of nutritionists and computer scientists at the Technical University of Munich. A survey on preferences, motives, and needs regarding nutritional information and digital gameplay was conducted among almost 300 children and adolescents before the game’s design [22]. The survey results were implemented in the game, which has been further evaluated within multiple usability tests and focus groups. An early description of the game is given elsewhere [23]. The FFF game presents itself as a journey through Europe, with each country focusing on country-specific food items. Every country includes three mini-games, which are described briefly as follows. The first mini-game is structured like a quiz that compares protein, fat, carbohydrates, or calories between two food items. The second mini-game is designed as an estimation game for the content of sugar, fat, or salt in one food item. Finally, the third mini-game is conceived as a physical activity unit. The user is instructed to pack a backpack with food and water in order to be sufficiently supplied with energy and liquids during the in-game activity. Moreover, bonus points are awarded for collecting at least five apples during the physical activity unit. Finally, after winning all three mini-games within one country, the next country is unlocked to travel and to explore. Besides the implicit display of the nutritional rules (German Nutrition Society) during gameplay, one guideline is also shown to participants before starting each mini-game. The content of the game is structured according to pedagogical models [24,25]. The tasks refer to food items from the previous country and provide different levels of achievement and motivational feedback. The gameplay intervention was carried out during school lessons. Due to the limited availability of tablets (Samsung Tab S2 T813; Android 7.0; Samsung, Seoul, South Korea), students were divided into six sub-groups and played the FFF game individually for 15 min on each of the three consecutive days. At least two study assistants were present to provide supervision and technical advice. However, no teachers were present during the gameplay intervention.

#### 2.2.2. Teaching Group

In the teaching condition, the study assistant taught students using a blackboard in their classrooms for about 15 min on each of the three consecutive days. The teaching content was based on similar content to the mini-games. However, interactive elements (quizzes) were also included. At the beginning of each teaching unit, two of the six selected rules of the German Nutrition Society were written one by one on the blackboard. After each rule was written out, students were asked to read the rule carefully and to explain the rule with their own words. Finally, students were asked nutritional quiz questions and were encouraged to answer them openly. During the teaching intervention, one teacher was always present. Both intervention modes (gameplay and teaching) were designed and executed by the same study assistant.

### 2.3. Measures

#### 2.3.1. Anthropometry

Body height and weight were measured at baseline by two study assistants. Weight was measured using a digital flat scale (seca 803; seca GmbH & Co. KG, Hamburg, Germany) to the nearest 0.1 kg. Height was assessed using a mobile stadiometer (seca 214; seca GmbH & Co. KG, Hamburg, Germany) to the nearest 0.1 cm. Measurements were performed in a standardized manner, without shoes but fully clothed. Each of the two study assistants had to confirm the accuracy of measurements performed. Body mass index (BMI) percentiles were used for the classification of children’s and adolescents’ weight status. Calculations were carried out using “my BMI 4 Kids” (https://aga.adipositas-gesellschaft.de/mybmi4kids/), an online tool provided by the German Working Group of Obesity in Childhood and Adolescence (AGA) [26]. Since participants’ birth dates were not available, the calculation of percentiles was based on life years only.

#### 2.3.2. Questionnaire

The pre- (baseline) and post-questionnaires were developed by an interdisciplinary research team of nutritionists and computer scientists, and covered both lifestyle- and game-related questions. The lifestyle section focused on knowledge, behavior, and attitudes regarding nutrition and physical activity, whereas the game section included questions about motivation, personality, and system usability (not further addressed hereafter). The primary outcome “knowledge” was assessed with the same questions (twelve single choice questions, six cloze sentences, and one open question) at pre- and post-intervention. The cloze sentences included four blanks each, where students had to fill in the answers. In general, questions referred to energy, macronutrients, fruits and vegetables, sugar and salt, water, and beverages, as well as physical activity (Table 1). Dependent on the question type, participants earned a different number of points. For each cloze sentence, one to a maximum of four points (rules: 6 × 4 points = 24 points) were achievable. A maximum of one point was possible for each of the other questions (miscellaneous nutrition and physical activity: 13 × 1 point = 13 points). Finally, the overall knowledge was assessed with a maximum achievable sum of 37 points.

Dietary behavior was assessed by a 45 item food frequency questionnaire (FFQ), taken from the Leipzig Lifestyle Questionnaire for Adolescents, 2007 [27], and covered eight food and five beverage groups. To assess dietary behavior, each food and beverage group was assessed by the following categories: adverse = 1, acceptable = 2, and optimal = 3. The resulting values (13–39) were used to calculate an overall dietary behavior score that represented a mean of the 13 single values. The resulting value for the overall dietary behavior score ranged from 1 to 3 and was interpreted according to above mentioned categories. Seasonal leisure time physical activity for summer and winter was evaluated on a scale (0, <1, 1–2, >2 h/week) according to Strobl et al. [28]. Based on the resulting scales for summer and winter, a physical activity score (no, moderate, or high) was calculated [28]. 

Finally, students’ attitudes towards healthy eating and the importance of the execution of a healthy diet were assessed with two single choice questions. Dietary behavior, physical activity, and healthy eating attitudes were assessed once at baseline.

### 2.4. Statistical Analyses

Accompanying the description of the participant characteristics (Table 2) as well as the healthy diet attitudes and physical activity behavior (Table 3), two-sample *t*-tests (metric variables) and chi-squared tests of independence (categorical variables) were performed to analyze possible differences between the GG and the TG.

To analyze changes in nutritional knowledge, paired *t*-tests were used to test for intervention effects within the groups. Two-sample *t*-tests were used to test the differences of the nutritional knowledge before (pre-) and after (post)-intervention between the GG and the TG. To explore possible explanatory confounding effects, linear regression models were fitted and adjusted for “gender”, “overall knowledge at baseline”, and “number of intervention days”. In the linear models, the differences between before (pre-) and after (post)-intervention were used as outcome variables. These linear regression models can be seen as adjusted versions of the two-sample *t*-tests for the detection of between-group differences. *p*-values < 0.05 were considered statistically significant. Analyses were conducted using the statistical software R (R Core Team 2018).

## 3. Results

### 3.1. Participant Characteristics

**Figure 1:**
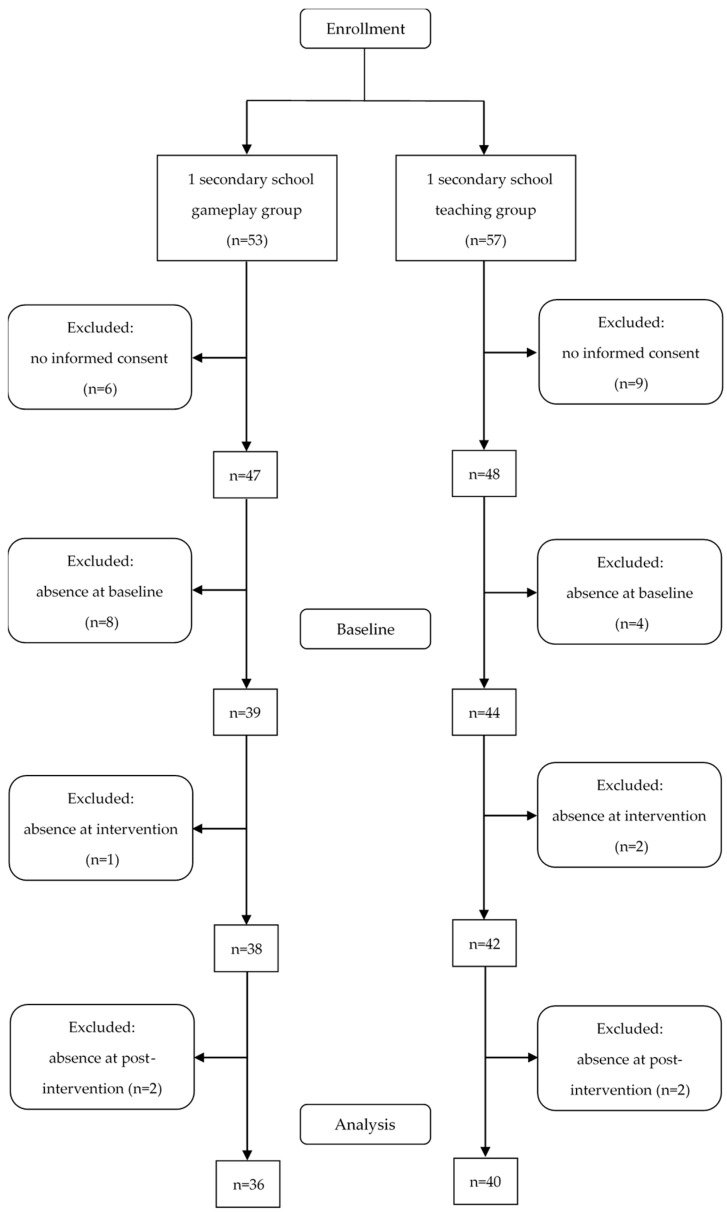
Flow diagram of study participants.

In total, 95 children and adolescents were enrolled in the study. After exclusion of participants with (1) missing baseline investigation, (2) missing post-intervention investigation, or (3) absence on all intervention days, data of 36 GG and 40 TG participants were analyzed (Figure 1).

Baseline socio-demographic and anthropometric characteristics of the study sample are shown in Table 2.

The mean age of the children and adolescents was 13.5 (± 0.7) years in the GG and 12.8 (± 0.9) years in the TG, with a statistically significant difference (*p* = 0.0003) between the groups. According to BMI percentiles, most of the GG participants (49%) and the TG participants (61%) had a normal weight. Fewer than five percent of participants were underweight (GG: 3% vs. TG: 2%). Overweight and (severe) obesity occurred in half of the GG participants (49%) and more than one-third of the TG participants (36%). Compared to the TG participants, severe obesity was three times more likely among GG participants (GG: 21% vs. TG: 7%). However, there were no statistically significant differences in BMI percentiles between both intervention groups (*p* = 0.5). Baseline data of children’s and adolescents’ attitude regarding a healthy diet and physical activity are presented in Table 3. The differences between both intervention groups were not statistically significant (*p* = 0.1; *p* = 0.4; *p* = 0.08).

In total, 67% (26/39) of participants in the GG and 84% (37/44) of participants in the TG reported that healthy foods were important to them. Furthermore, 62% (23/37) of the GG participants and 73% (32/44) of the TG participants stated that they pay attention to a healthy diet. Moreover, nearly 60% (22/38 and 27/42, respectively) of participants in both intervention groups showed a moderate physical activity score (Table 3). Compared to the GG, more participants in the TG showed a high level of physical activity (GG: 21% vs. TG: 31%). Moreover, GG participants had four times higher levels of low physical activity than TG participants (GG: 21% vs. TG: 5%). 

**Figure 2:**
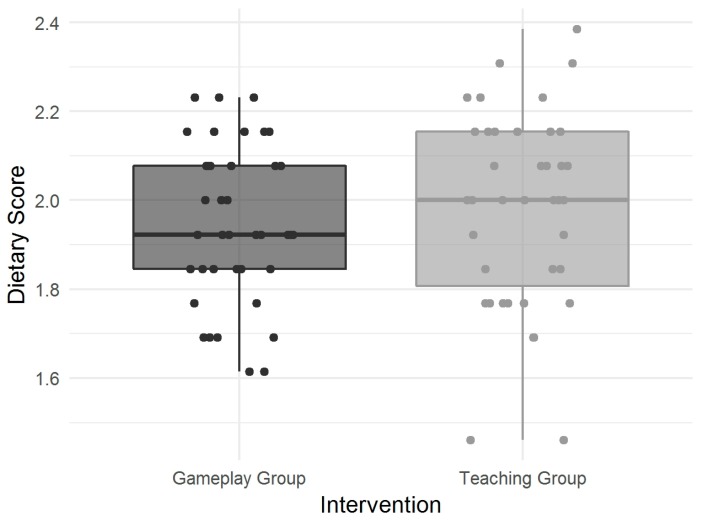
Dietary behavior at baseline depicted as overall dietary score.

According to the FFQ, most participants exhibited acceptable dietary behavior, with a mean dietary score of 1.9 (± 0.2) in the GG and 2.0 (± 0.2) in the TG at baseline (Figure 2).

### 3.2. Nutritional Knowledge

The changes in different categories of nutritional knowledge in both intervention groups are presented in Table 4.

Children and adolescents in both intervention groups showed improved knowledge from baseline to the post-intervention assessment. Total knowledge about the six rules of the German Nutrition Society increased significantly (*p* = 0.02) from 0.25 to 0.33 during the gameplay intervention, indicating that 25% of possible points were achieved at baseline and 33% at post-intervention. Moreover, total miscellaneous knowledge about nutrition and physical activity significantly improved within the GG from 0.43 to 0.57 (*p* < 0.0001). A significant change in nutritional knowledge was also detected for overall knowledge in the GG (0.31 vs. 0.42; *p* = 0.001). Similar results were found in the TG condition. The total knowledge of all categories (rules; miscellaneous; overall knowledge) significantly increased over time (*p* < 0.0001). Group differences between GG and TG were significant regarding total knowledge about nutritional rules (*p* = 0.02) and overall knowledge (*p* = 0.01), with a greater improvement in nutritional knowledge observed in the TG. The gender-specific analysis did not show significant differences in points between boys and girls in the change of overall knowledge in both intervention groups.

Results did not change after adjustment for “gender”, “overall knowledge at baseline”, and “number of intervention days”. As in the unadjusted model, the change of knowledge on the six rules of the German Nutrition Society (*p*-value = 0.02) as well as on overall knowledge (*p*-value = 0.02) remained significantly different between both intervention groups, with higher improvements in the TG.

## 4. Discussion

This study compared the short-term effectiveness of the serious game FFF on nutritional knowledge with traditional teaching in a sample of secondary school students. The results show that both interventions significantly improved nutritional knowledge in students, with higher improvement in the TG. Moreover, it was obvious that most students were at moderate levels of both dietary behavior and physical activity at baseline.

The significant improvement of knowledge in the TG was in line with results from other studies. Dudley et al. conducted a systematic review of 49 studies with teaching interventions among primary school children (up to 12 years) regarding healthy eating [29]. One-third of the studies evaluated the effect of teaching interventions on nutritional knowledge, and observed almost exclusively significant results. Besides traditional teaching strategies, innovative interventions (e.g., games) were covered as well [29]. Another systematic review of 16 empirical studies or reviews on traditional educational games (e.g., quizzes, card and board games) for classroom education revealed that both traditional didactic methods and game-based educational strategies are effective in imparting nutritional knowledge, with no clear advantages for one strategy [6]. Moreover, authors reported that research is still limited, and concluded that games might be more beneficial for the long-term retention of information due to their enjoyable and entertaining nature [6]. Amaro et al. examined the efficacy of the board-game “Kalèdo” with regard to nutritional knowledge, dietary behavior, and physical activity among more than 300 secondary school students aged from 11 to 14 years [30]. While the game group played “Kalèdo” 15 to 30 min once a week, the control group received no intervention. Compared to controls, the game group significantly improved nutritional knowledge (*p* < 0.05) and dietary behavior (*p* < 0.01; vegetable intake) after 24 weeks [30]. These results were reproduced among a sample of more than 3000 children and adolescents (9 to 19 years) for a six month intervention period [31]. This study revealed that the game group showed significantly improved nutritional knowledge compared to the non-treatment control group [31]. In summary, traditional classroom teaching with and without gamification elements might be successful for the improvement of nutritional knowledge.

In the present study, the GG significantly improved knowledge during a three day intervention, with a significant difference compared to the TG. Results were in line with data from a study by Hermans et al. among 108 elementary school children aged from 10 to 13 years [32]. Participants were assigned either to play the nutritional game “Alien Health Game” (AHG) or to play the web-based nutritional game “Super Shopper” (SS) for half an hour on each of two consecutive days. Compared to baseline, the AHG had significantly higher improvements in nutritional knowledge (*p* < 0.001) than the SS group at immediate post-test. This effect was not maintained for the follow-up period of two weeks. Moreover, there was no evidence for behavior change [32]. As both digital and analogue games have shown efficacy in imparting nutritional knowledge, it might be assumed that gamification itself is beneficial regarding educational purposes.

In the context of the present study, the teaching sessions included both blackboard teaching and interactive elements (quizzes). Therefore, it might be possible that the combination of both elements was beneficial for the improvement of nutritional knowledge. During the teaching sessions, students focused their attention on the study assistant and the blackboard where the nutritional rules of the German Nutrition Society were presented. This teaching method may have been more effective in directing students’ attention toward the educational content compared to students in the GG, who did not receive personal instruction and could have been distracted by the game environment. Although students of the GG had to confirm digitally that they read the displayed nutritional rules, it cannot be ensured that this was done. In contrast to the gameplay session, teachers were present during the teaching units, which might have influenced the students’ attention. Moreover, the study assistant who did the classroom teaching was not blinded and was involved in the study design. In contrast to the gameplay intervention (no personal interaction), the teaching intervention was performed face-to-face. Therefore, it could be assumed that participants were influenced unconsciously and unintentionally by the study assistant (e.g., appearance, facial expressions, and gestures).

It is questionable whether a daily 15 min intervention for three days can affect nutritional knowledge among children and adolescents in the long-term. A literature review on nutrition-related gamification revealed that most studies have focused on short-term experiments [33]. To assess the long-term effectiveness of gamification interventions on dietary behavior changes, longitudinal studies are necessary [33]. Extending the intervention period and the frequency of the FFF gameplay may induce long-term effects not only on knowledge, but also on dietary behavior. Furthermore, research indicates that playing digital games can be affected by high attrition [20,34,35], which calls into question whether greater effects would have been observed within longer intervention periods. Although students showed improved nutritional knowledge, the change from pre- to post-intervention was rather small, with participants achieving fewer than 50% of the maximum number of points. Therefore, it is unknown whether this level of knowledge improvement might have a practical impact on nutritional knowledge and behavior.

This pilot study had some limitations. First, the study population was not representative for the general population of German children and adolescents, due to its small and rather homogenous sample size (secondary schools). Future research should address more diverse populations (different school types, social environments, and regions). Second, the recruitment of students was the class teachers’ responsibility. Thus, the response rate might have been influenced by the personal characteristics (e.g., attitude and motivation) of the teachers. Furthermore, parents might also have biased the participation rate, since children required parental consent to participate. Moreover, a selection bias might be present, as it is likely that only students who were interested in the research topics (nutrition and digital games) participated. Additionally, the proportion of male participants was large in both intervention groups, which was caused by a high proportion of boys within the participating classes. Therefore, the male gender was slightly overrepresented within the present pilot study. Furthermore, the total number of participants differed between the intervention groups as well as between the variables.

Finally, studies with extended intervention periods might enable researchers to assess the impact of a digital nutritional game and classroom teaching on outcomes such as dietary behavior and body weight. According to the recommendations for a sound game design within the literature, the FFF game was designed by an interdisciplinary team of nutritionists and computer scientists, ensuring an evidence-based development and evaluation process. Furthermore, formative research (surveys and focus groups) was conducted among the target group before and during the game design [22,23].

## 5. Conclusions

The results of this pilot study showed that both short-term educational interventions increased nutritional knowledge in children and adolescents, with larger improvement in the TG compared to the GG. Since nutritional knowledge might be deficient in this age group and digital gameplay is popular among young people, a serious game might be an adequate tool to impart nutritional knowledge in an entertaining format. There is limited evidence for the effectiveness of serious games for nutritional education, especially in Germany. Moreover, it is still unclear whether nutritional knowledge can translate to dietary behavior. Therefore, further research is warranted to evaluate the long-term effectiveness of serious games on nutritional knowledge and dietary behavior.

## Figures and Tables

**Table 1:** Overview of questions related to knowledge of nutrition and physical activity.

Nr	Parameter	Question
**Selected Rules of the German Nutrition Society**		
1	Select (*whole grain*)^1^: For cereal products such as bread, pasta, rice, and flour, the (*whole grain*) choice is the best choice for your health. Foods made from (*whole grain*) satiate longer and contain (*more*) nutrients than white flour products.	Cloze
2	Reduce (*sugar*) and salt intake: Food and beverages sweetened with (*sugar*) are (*not*) recommended. Avoid them as much as possible and use (*sugar*) sparingly. Reduce salt and reduce the amount of salty food.	Cloze
3	Choose health-promoting fats: Prefer (*vegetable*) oils such as rapeseed oil and spreadable fats made from it. Avoid (*hidden*) fats. Fat is often “invisible” in processed foods such as (*sausage*), pastries, sweets, fast food, and (*convenience products*).	Cloze
4	Pay attention to body weight and stay (*active*): A wholesome (*diet*) and physical (*activity*) belong together. Not only regular sport is important, but also an active daily life. Per day, 30 to (*60*) min of moderate physical activity promotes your health.	Cloze
5	Choose (*water*) as your drink of choice: Drink around (*1.5*) liters every day. Ideally (*water*) or other non-caloric drinks such as unsweetened tea. Sugar-sweetened and alcoholic beverages are (*not*) recommendable.	Cloze
6	Vegetables and (*fruits*)—choose “5 a day”: Enjoy at least (*3*) portions of vegetables and (*2*) portions of (*fruits*) each day.	Cloze
**Miscellaneous—Nutrition**		
7	What is the meaning of the term “calories” and what is a different term for calories?	Single choice
8	What is another word for protein?	Open
9	Is sugar a carbohydrate?	Single choice
10	How many servings of fruits and vegetables should you eat daily (one serving = handful)?	Single choice
11	Which of the following statements is correct? In daily nutrition you should... Reduce sugar, but you can eat as much salt as you like OR Use salt sparingly, but you can eat as much sugar as you like OR Reduce neither salt nor sugar OR Reduce sugar and salt OR Don’t know	Single choice
12	Which of the following statements is correct? Whole grain bread contains more nutrients (e.g., fiber) than white bread AND Satiates more than white bread OR White bread contains more nutrients (e.g., fiber) than whole grain bread OR White bread and whole grain bread do not differ in terms of nutrients (e.g., fiber) AND Satiety OR Don’t know	Single choice
13	Which of the following foods has the highest fat content for the same amount? Chocolate OR Hard caramels/sweets OR Jelly Bears OR Cereal bar OR Marshmallows OR Don’t know	Single choice
14	Which of the following statements is correct? Fruits and vegetables contain the same amount of sugar OR Vegetables contain less sugar than fruits for the same amount OR Fruits contain less sugar than vegetables for the same amount OR Don’t know	Single choice
15	Which of the following foods has the highest sugar content for the same amount? Soft drinks (e.g., lemonade, cola) OR Water OR Cow’s milk OR Juice spritzer OR Tomato juice OR Don’t know	Single choice
16	Which of the following statements is correct? Protein is particularly high in...Clarified butter OR Cucumber OR Pizza OR Licorice OR Tuna fish OR Don’t know	Single choice
17	What does the abbreviation DGE stand for? German Cooperative for Nutrition OR German Society for Global Warming OR German Society for Nutritional Habits OR German Nutrition Society OR Don’t know	Single choice
**Miscellaneous—Physical Activity**		
18	How many calories (kcal) do you expend approximately if you ride a bike at medium effort for 2 h? Less than 200 kcal OR 600–1000 kcal OR More than 1000 kcal OR Don’t know	Single choice
19	In which of the following three sports do you expend the most energy during the same performance (e.g., duration, distance)? Cycling OR Running/Jogging OR Swimming OR Don’t know	Single choice

^1^ Italic words in parentheses represent the gaps within the cloze sentences.

**Table 2:** Socio-demographic and anthropometric characteristics of intervention groups at baseline.

Parameter	Gameplay Group (GG)			Teaching Group (TG)			Difference
	Total N (%) or mean ± SD	Female N (%) or mean ± SD	Male N (%) or mean ± SD	Total N (%) or mean ± SD	Female N (%) or mean ± SD	Male N (%) or mean ± SD	p
Total	39	13	26	44	12	32	
7th grade	27	10	17	28	8	20	
8th grade	12	3	9	16	4	12	
Age (y)	13.5 ± 0.7	13.4 ± 0.8	13.6 ± 0.8	12.8 *±* 0.9	12.5 ± 0.9	12.9 *±* 0.9	0.0003
Height (m)	1.66 ± 0.1	1.60 ± 0.06	1.69 ± 0.09	1.61 *±* 0.08	1.60 ± 0.06	1.61 ± 0.09	0.006
Weight (kg)	70.0 ± 18.6	70.3 ± 22.8	69.8 ± 16.6	57.8 ± 14.7	60.7 ± 15.9	56.7 ± 14.3	0.001
BMI percentiles							0.5
Underweight	1 (3%)	1 (8%)	0 (0%)	1 (2%)	0 (0%)	1 (3%)	
Normal weight	19 (49%)	5 (38%)	14 (54%)	27 (61%)	7 (58%)	20 (62%)	
Overweight	6 (15%)	1 (8%)	5 (19%)	8 (18%)	2 (17%)	6 (19%)	
Obesity	5 (13%)	0 (0%)	5 (19%)	5 (11%)	1 (8%)	4 (12%)	
Severe Obesity	8 (21%)	6 (46%)	2 (8%)	3 (7%)	2 (17%)	1 (3%)	

N = number; SD = standard deviation; p = *p*-value; yrs = years; m = meter; kg = kilogram; BMI = body mass index; underweight: BMI percentile < 10; normal weight: BMI percentile 10–90; overweight: BMI percentile > 90–97; obesity: BMI percentile > 97–99.5; severe obesity: BMI percentile > 99.5.

**Table 3:** Healthy diet attitudes and physical activity at baseline.

Parameter	Gameplay Group (GG)		Teaching Group (TG)		Difference
	n/N	%	n/N	%	p
Healthy diet “importance”	26/39	67	37/44	84	0.1
Healthy diet “attention”	23/37	62	32/44	73	0.4
Physical activity					0.08
*High*	*8/38*	*21*	*13/42*	*31*	
*Moderate*	*22/38*	*58*	*27/42*	*64*	
*Low*	*8/38*	*21*	*2/42*	*5*	

n/N = number; p = *p*-value.

**Table 4:** Changes in knowledge from pre- (baseline) to post-intervention by and between groups.

Knowledge	Gameplay Group (GG)				Teaching Group (TG)				Difference
Category	N	Pre	Post	p	N	Pre	Post	p	p
**Rules**	25				40				
(Whole) grain		0.08	0.16			0.14	0.23		
Sugar & salt		0.26	0.35			0.35	0.48		
Fats & oils		0.12	0.10			0.03	0.08		
Lifestyle		0.21	0.22			0.15	0.31		
Water & beverages		0.47	0.63			0.38	0.62		
Vegetables & fruits		0.52	0.53			0.42	0.65		
*Total knowledge*		*0.25*	*0.33*	*0.02*		*0.24*	*0.39*	*<0.0001*	*0.02*
**Miscellaneous**	35				40				
Nutrition		0.44	0.58			0.40	0.58		
Physical activity		0.39	0.53			0.31	0.50		
*Total knowledge*		*0.43*	*0.57*	*<0.0001*		*0.39*	*0.56*	*<0.0001*	*0.3*
**Overall knowledge**	24	0.31	0.42	0.001	40	0.29	0.45	<0.0001	0.01

N = number; p = *p*-value; Difference is given by the proportion of achieved points relative to the maximum of achievable points. The “total knowledge” refers to the respective category (e.g., rules). The category “overall knowledge” is the combination of the total knowledge “rules” and the total knowledge “miscellaneous”.
